# Evidence for a Shared Etiological Mechanism of Psychotic Symptoms and Obsessive-Compulsive Symptoms in Patients with Psychotic Disorders and Their Siblings

**DOI:** 10.1371/journal.pone.0125103

**Published:** 2015-06-10

**Authors:** Marije Swets, Frank Van Dael, Sabine Roza, Robert Schoevers, Inez Myin-Germeys, Lieuwe de Haan, Genetic Risk and Outcome of Psychosis (GROUP) investigators

**Affiliations:** 1 Arkin Mental Health and Addiction Treatment Centre, Amsterdam, the Netherlands; 2 Department of Psychiatry and Neuropsychology, South Limburg Mental Health Research and Teaching Network, EURON, Maastricht University, Maastricht, the Netherlands; 3 Department of psychiatry, Erasmus University Medical Center, Erasmus University, Rotterdam, the Netherlands; 4 Department of psychiatry, University Medical Centre Groningen, University of Groningen, Groningen, the Netherlands; 5 Academic Medical Centre University of Amsterdam, Department of Psychiatry, Amsterdam, The Netherlands; UTHSCSH, UNITED STATES

## Abstract

The prevalence of obsessive-compulsive disorder in subjects with psychotic disorder is much higher than in the general population. The higher than chance co-occurrence has also been demonstrated at the level of subclinical expression of both phenotypes. Both extended phenotypes have been shown to cluster in families. However, little is known about the origins of their elevated co-occurrence. In the present study, evidence for a shared etiological mechanism was investigated in 3 samples with decreasing levels of familial psychosis liability: 987 patients, 973 of their unaffected siblings and 566 healthy controls. The association between the obsessive-compulsive phenotype and the psychosis phenotype c.q. psychosis liability was investigated. First, the association was assessed between (subclinical) obsessive-compulsive symptoms and psychosis liability. Second, in a cross-sib cross-trait analysis, it was examined whether (subclinical) obsessive-compulsive symptoms in the patient were associated with (subclinical) psychotic symptoms in the related unaffected sibling. Evidence was found for both associations, which is compatible with a partially shared etiological pathway underlying obsessive-compulsive and psychotic disorder. This is the first study that used a cross-sib cross-trait design in patients and unaffected siblings, thus circumventing confounding by disease-related factors present in clinical samples.

## Introduction

Obsessive compulsive disorder (OCD) and obsessive compulsive symptoms (OCS) in schizophrenia are a cause of significant suffering [1] and often remain undiagnosed [2], even though successful treatment for this comorbid condition has been reported [3]. The prevalence rate of comorbid OCD in schizophrenia is over ten times higher than the prevalence for OCD of 1.0% found in the community [4] [5]. A recent meta-analysis yielded mean average prevalence rates of 13.6% for comorbid OCD, 20.5% for OCS defined as a score on the Yale-Brown Obsessive-Compulsive disorder Scale (YBOCS) of >9 and 30.3% for *any* comorbid OCS (YBOCS>0) [6]. Patients with schizophrenia often have co-morbid conditions [7], but the difference in prevalence of OCS in schizophrenia patients compared to the base rate prevalence in the general population is higher than the difference in prevalence of other anxiety disorders and depression [8, 9]. The origin of this high co-occurrence is largely unknown, but some recent findings give reason to examine the evidence for a partially shared pathogenetic mechanism in the development of these two disorders. Mainly due to genetic influences, siblings of patients with schizophrenia have a 5- to 10-fold higher risk to develop schizophrenia [10]. Research has shown that schizotypy and (subclinical) psychotic symptoms cluster in families, and are likely to be part of an extended psychosis phenotype [11–13]. However, little is known about how this liability to psychosis relates to liability to OCS. Other causal mechanisms are also proposed, some of which may be disease-related or treatment-related factors, such as the exposure to antipsychotic medication [14–17].

One method to disentangle the contribution of these mechanisms is to investigate the occurrence of OCS in the unaffected siblings of patients with psychosis, thus ruling out the role of disease-related factors. To our knowledge, only one family study investigated relatives of patients with schizophrenia with and without OCD [18]. Relatives of patients with combined schizophrenia and OCD showed significantly increased rates of schizophrenia with co-morbid OCD or obsessive-compulsive personality disorder in comparison to relatives of patients with non-OCD schizophrenia. However, it was not clear whether the risk of OCS was higher in relatives of patients with non-OCD schizophrenia. Furthermore, a substantial portion of the subjects was not interviewed directly, analyses did not control for the fact that data were paired, data of siblings and parents were pooled together and no direct associations between siblings and patients were analysed.

The current study is using the GROUP sample, a large and representative cohort of patients, their non-psychotic siblings (hereafter referred to as “siblings”) and healthy controls not related to first or second degree family members with psychotic disorders, to investigate evidence for a familially shared and possibly common genetic etiology between psychosis and OCD.

First, the association between (subclinical) OCS and psychosis liability was examined by using status (patient, sibling or control) as a measure for psychosis liability. If OCS and psychotic symptoms have a common etiology that is shared in families, unaffected siblings would have an elevated risk for the development of OCS compared to controls. If this association remains when controlling for the expression of (subclinical) psychotic symptoms, the indication for a shared familial-possibly genetic- etiology would even be stronger.

As OCD itself clusters in families [19–21], subclinical OCS in siblings of patients with OCD may reflect a familial OCD liability. OCD liability may exist independent of psychosis. In order to diminish the role of such an independent familial OCD liability from the examined association between OCS and psychosis liability, siblings belonging to families with patients with OCD (defined as YBOCS score > = 16) [22–24] were excluded from this analysis.

Next, it was examined, using cross-sib within-trait analyses, whether the clustering of OCS within families shown in the general population, could also be found within families with psychotic disorder.

Finally, cross-sib cross-trait analyses were conducted to investigate whether OCS in one sibling were associated with psychosis levels in the other sibling. The fact that OCS in patients are associated with psychosis dimensions in unaffected siblings would support the hypothesis of a familially shared, possibly genetic etiology.

## Methods

### Subjects

Data were collected from the Genetic Risk and Outcome in Psychosis study (GROUP) [25] *(data are available upon request)*. In representative geographical areas in the Netherlands and Belgium, patients were identified through clinicians working in regional psychotic disorder services, whose caseload was screened for inclusion criteria. Subsequently, a group of patients presenting consecutively at these services either as out-patients or in-patients and their siblings were recruited for the study. Controls were selected through a system of random mailings to addresses in the catchment areas of the cases. Inclusion criteria for patients were: (i) age range 16 to 50 years, (ii) diagnosis of non-affective psychotic disorder and (iii) good command of Dutch language. Controls had no first degree relative with a psychotic disorder as established by the Family Interview for Genetic Studies with the control as informant [26]. Diagnosis was based on the Diagnostic and Statistical Manual of Mental Disorder-IV (DSM-IV) criteria [27], assessed with the Comprehensive Assessment of Symptoms and History (CASH) interview [28] or Schedules for Clinical Assessment for Neuropsychiatry (SCAN 2.1) [29]. In case a sibling made a transition to a psychotic illness, he or she was allocated to the patient cohort. The current study focused on the patients, siblings and controls in this sample.

This study was approved by the standing ethics committees of the University Medical Centres of Utrecht (GROUP study), Amsterdam, Maastricht and Groningen. All subjects gave written informed consent in accordance with the committee’s guidelines. The committees waived the need for additional informed consent of parents or supervisors for underaged participants ages 16 and older, given the non-experimental/medical nature of this study.

### Measurement of compulsive and obsessive symptoms

An important challenge facing clinicians and researchers is differentiating an obsession from delusion. We used a “conservative approach”: OCS was defined according to the Structured Clinical Interview for DSM-III-R patient-edition (SCID-P) as persistent, repetitive, intrusive, and distressful thoughts (obsessions) not related to the subject’s psychotic symptoms or repetitive goal directed rituals (compulsions) clinically distinguishable from mannerisms or posturing observed in schizophrenia. Consequently, patients whose obsessions or compulsions were related to psychotic content of thoughts, or patients without insight in their obsessions or compulsions, were not diagnosed with co-morbid OCS in this study. Insight was assessed by the interviewer. To measure obsessions and compulsions the Yale-Brown Obsession and Compulsion Scale (Y-BOCS) was used. The Y-BOCS is a 10-item semi-structured interview designed to measure the severity of OCS over the previous week [30, 31]. When an OCS is present, its severity is measured with five items. These items address time spent on, interference and distress from, resistance against and perceived control over obsessions or compulsions, rated on a 5 point scale (0–4). The Dutch translation of the Y-BOCS [32] was used in this study. A broad definition of OCS (hereafter called *OCS*), meaning any symptom present, was defined as a Y-BOCS score of 0 versus >0. In addition, clinically relevant OCS were defined as a Y-BOCS cut-off of >9 (value 1) or < = 9 (value 0) (hereafter *clinically relevant OCS*) [33, 34].

### Psychosis measures

Psychotic experiences were measured using an interview measure-the Structured Interview for Schizotypy-Revised (SIS-R) [35, 36]. The SIS-R is an elaborate semi-structured interview measuring subclinical psychotic experiences in non-psychotic subjects, conducted in siblings and controls. Based on results of previous research on the SIS-R, a positive symptom and negative symptom dimension score was calculated, representing the means of the positive schizotypy item scores and of the negative schizotypy scores respectively. Because the distribution of the SIS-R scores was skewed, it was divided by its deciles, in order to create decile groups.

### Analyses

All statistical analyses were performed using SPSS 17.0 or STATA version 12. Given that some families contributed more than 1 sibling, hierarchical clustering of data at the level of family was modelled using the multilevel random regression XTMELOGIT or XTMIXED routine in STATA version 12 statistical software [37]. Pearson Chi-Square tests, T-test and logistic regression were used when appropriate, in order to determine demographics, illness characteristics and prevalences. Estimates were adjusted for a priori determined confounders (sex, age, ethnicity, marital status (single or not), education level and IQ).

#### Association between OCS and status

The first approach was to investigate the association between OCS and genetic psychosis liability, operationalized as a three-level categorical variable “status” (0 = controls, 1 = unaffected siblings, 2 = patients with a psychotic disorder). The association between OCS and status was estimated using multilevel logistic regression analysis (xtmelogit) expressed in odds ratios.

In the next step, the analyses were restricted to siblings and the controls, thereby excluding possible confounding by disease-related factors. Associations between OCS and the two-level variable quantifying psychosis liability (sibling versus controls) were investigated. As mentioned in the introduction, siblings belonging to families with patients with OCD (defined as YBOCS score > = 16) were excluded from this analysis. Associations were adjusted for phenotypically expressed subclinical positive and negative psychotic symptoms, measured with the SIS-R. In case of significant associations, the model was adjusted for the *a priori* determined confounders. These analyses were repeated excluding the siblings of patients with OCD (corresponding with a Y-box score > = 16, in line with earlier studies [38].

#### Cross-sib within trait and cross-sib cross-trait associations

**Fig 1:**
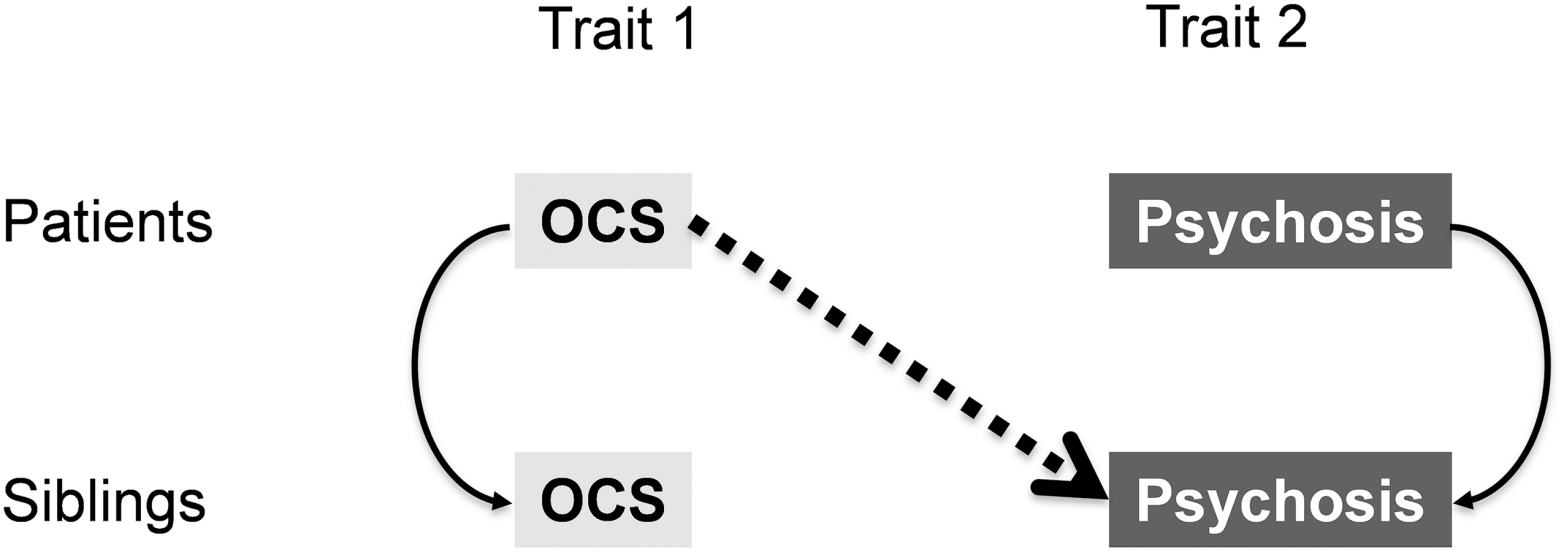
Cross-sib cross-trait design. Is one trait in one sib associated with another trait in the other sib?

Second, it was examined whether familial clustering of the OCD phenotype shown in the general population was also encountered in a sample with psychosis liability, and, additionally, whether clustering of OCD and psychosis phenotypes could be detected. For this purpose, the cross-sibling within-trait associations (association of trait x in sibling A (patient) with the same trait x in related sibling B (sibling)) and cross-sibling cross-trait associations (the association of trait x in sibling A (patient) with trait y in related sibling B (sibling)) were investigated (Fig 1). In the case of families contributing more than 1 patient or more than 1 sibling, all possible patient–unaffected sibling pairs were included in the analyses.

We started to investigate whether a cross-sib within OCS trait association could be confirmed in this sample, by regressing the dichotomous YBOCS score (> = 16 vs. <16) in the unaffected sibling on that of the related patient using the XTMELOGIT routine in STATA version 12 statistical software [37]. Next, it was examined whether OCS and psychotic symptoms were associated across siblings. Therefore, the SIS-R scores of unaffected relatives were regressed on the dichotomous YBOCS scores in patients using the XTMIXED routine. Multilevel modelling was used as some families contributed more than 1 sibling. Effect sizes were expressed as coefficient b from linear regression analyses. In case of a significant association, the model was adjusted for the *a priori* determined demographical confounders (sex, age, ethnicity, marital status (single or not), level of education, IQ) and next for use of antidepressant and antipsychotic agents.

## Results

### Sample characteristics

A total of 3,686 persons was included in the GROUP-study. Of these, 1,120 (30.4%) were patients coming from 1,060 families. A total of 1,976 relatives was included (53.6% of total sample), coming from 919 families. The control group consisted of 590 healthy persons coming from 494 non-affected families. The total number of families included in the study was 1,561, with a maximum of 8 included relatives per family. Patients with missing diagnoses (and their relatives) were excluded from the present study (6 patients, 7 relatives). Of the remaining 3,673 persons, 297 (8.1%) did not have valid information on the presence or absence of obsessive-compulsive symptoms and were also excluded. All 850 parents were excluded. Overall, 2,526 subjects were included in one or more analyses (987 patients and 973 siblings, coming from 978 families, and 566 controls) (Table 1).

**Table 1:** Distribution of 2526 subjects in 1458 families.

	(patients + sibs)	Patients	Siblings	Controls
2526 (1458)				
Fam. with psychosis liability	1960 (978)	987 (934)	973 (770)	-
Gen. Pop. controls	-	-	-	566 (480)
Fam. with OCD pts.	169 (83)	91 (83)	78 (65)	-
Fam. without OCD pts.	1740 (851)	896 (851)	844 (661)	-
Fam.without pts.*	51 (44)	-	51 (44)	-

Notation: number of subjects in (number of families)

* families without patients with sufficient valid ybocs scores.

### Demographic and basic clinical characteristics

Descriptives of the total sample are presented in Table 2. Compared to controls, patients were more often of male sex (χ^2^(1) = 151, p < 0.001), younger (t(1) = 5.5, p < 0.001), of lower educational level (χ^2^(3) = 143, p < 0.001), single (χ^2^(2) = 209, p < 0.001), and of non-white ethnic group (χ^2^(1) = 40.4, p < 0.001); patients also had a lower IQ (t(1) = 17.5, p < 0.001) and more often used cannabis (χ^2^(1) = 47.7, p < 0.001). Sibs were younger (t(1) = 5.4, p < 0.000), of lower educational level (χ^2^(3) = 42, p < 0.000), of non-white ethnic group (χ^2^(1) = 14.5, p < 0.000); they also had a lower IQ (t(1) = 9.0, p < 0.000) and more often used cannabis (χ^2^(1) = 7.3, p < 0.007) than controls, but showed no significant differences in sex (χ^2^(1) = 0,04, p = 0.8) and relationship status (single or with partner) (χ^2^(2) = 0.0, p = 1.0).

**Table 2:** Descriptives of study population.

	Patients (n = 987)	Siblings (n = 973)	Controls (n = 566)
Sex (% male)	76.3	45.9	45.4
Age (mean, sd)	27.8 (8.2)	27.8 (8.3)	30.4 (10.6)
*Educational level*			
% < = primary	12.7	7.8	2.8
% secondary (VMBO/HAVO/MBO)	59.8	51.8	43.1
% higher (VWO/HBO/WO)	25.2	38.4	53.4
*Marital status*			
% married/living together	9.1	49.9	39.8
Ethnicity (% White)	77.2	83.0	90.1
Cannabis in urine (% positive)	16.6	8.0	4.3
IQ (mean, sd)	95.0 (16.0)	102.3 (15.5)	109.7 (15.2)
Quality of life (% bad)	14.9	3.6	1.6
**Patient descriptives**			
GAF(mean, sd)	56 (15.9)		
*Diagnosis (number*, *% of patients)*			
Schizophrenia	659 (66,8%)		
Schizophreniform disorder	51(5,2%)		
Other psychotic disorder	156(15,8%)		
Mood component (bipolar, schizoaffective, depressive)	121 (15.8%)		
Duration of illness (median, 95% range)	3.6 (0.2–15.1)		
Age at onset (mean, sd)	22.3 (6.8)		
Number of psychotic episodes (100% range)	1–8		
*Current use of antipsychotic medication (%)*			
No or unknown	29.6		
Classic antipsychotics	8.1		
Risperidon/quetiapine/aripiprazole	28.6		
Clozapine	7.7		
Olanzapine	21.4		
Combinations	4.5		

### Association between OCS and status

Broadly defined *OCS* (Y-BOCS >0) were significantly more prevalent in patients (22.7%, n = 224) (OR = 6.7, 95% CI:4.3–10.6, p<0.000) and siblings (8.2%, n = 80) (OR = 1.8, 95% CI:1.1–3.0, p<0.012) compared to controls (4.8%, n = 27). Patients reported significantly more OCS compared to siblings (χ^2^(1) = 70.4,p<0.000).

**Fig 2:**
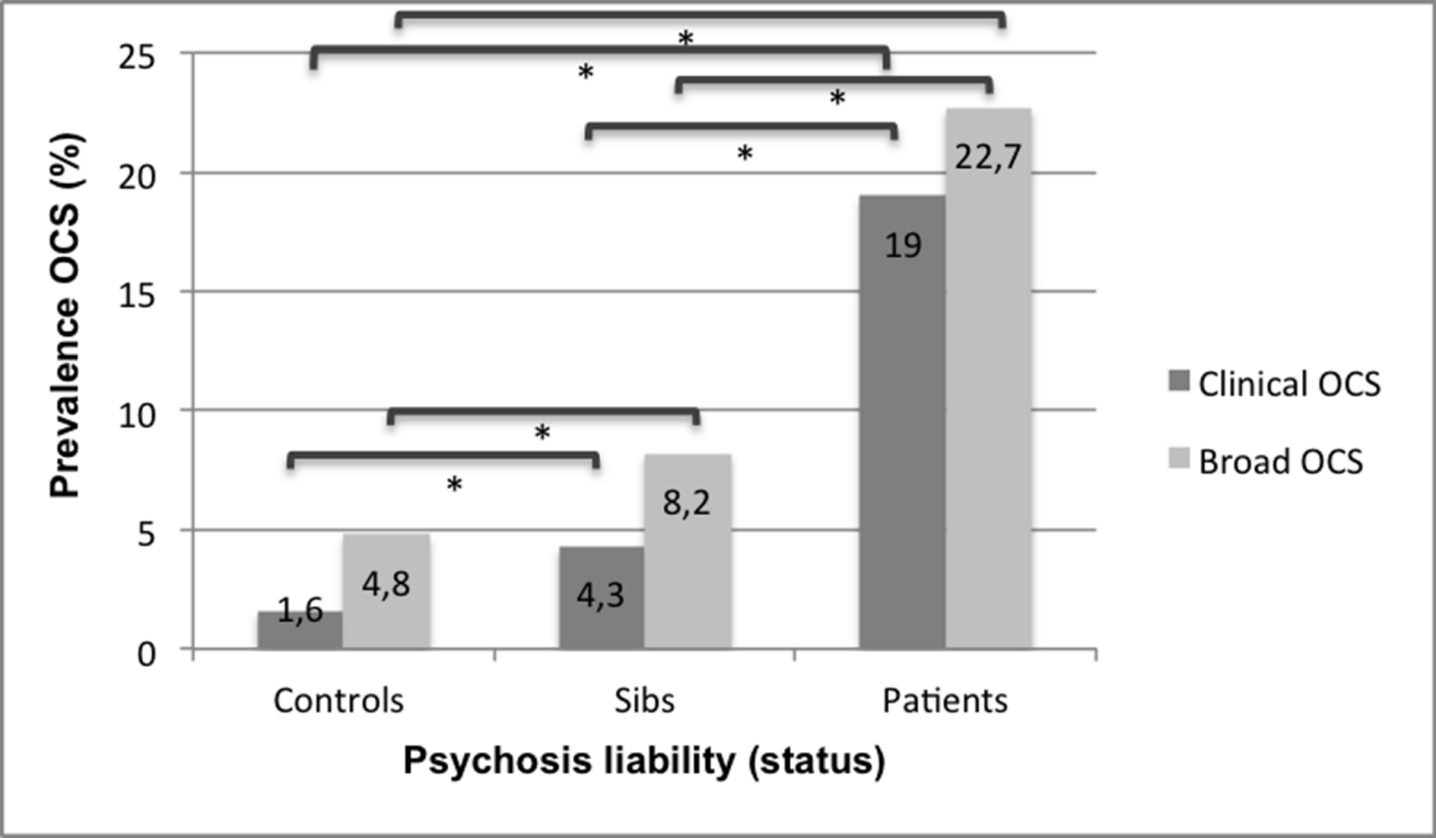
Prevalence of OCS in function of 3-level psychosis liability (status). Clinical OCS: YBOCS>9. Broad OCS: YBOCS>0. * Differences in prevalence between distinct psychosis liability groups are all significant.

*Clinically relevant OCS* (Y-BOCS >9) were significantly more prevalent in patients (19.0%, n = 188) (OR = 18.4, 95% CI:8.8–38.7, p<0.000) and siblings (4.3%, n = 42) (OR = 2.9, 95% CI:1.4–6.2, p<0.006) compared to controls (1.6%, n = 9). Patients reported significantly more clinically relevant OCS compared to siblings (χ^2^(1) = 80.6,p<0.00005). The associations remained significant after adjusting for confounders (Fig 2).

Clinically relevant OCS (YBOCS>9) were present in 10.3% of the siblings of patients with co-occurring OCD and psychotic disorder, compared to 4.0% in the siblings of patients with psychotic disorder without OCD and 1.6% in controls. The latter difference was significant, using a t-test (p<0.01). Furthermore, in the unaffected siblings of patients with psychotic disorder but without OCD, clinically relevant OCS were significantly associated with psychosis liability.

In the next step, analyses were restricted to siblings and controls. It was examined whether the association between OCS and two-level status (sibling versus controls, or sibling status) remained significant when adjusted for positive and negative subclinical psychotic symptoms as assessed with the SIS-R. The association between OCS and two-level status remained significant (broadly defined OCS: OR = 1.7, 95% CI: 1.0–2.6, p<0.032; clinically relevant OCS: OR = 2.7, 95% CI:1.3–5.9, p<0.01). Additional adjustment for the confounders did not substantially alter the results. When adjusted for a diagnosis of depression in the subsample with CASH data (n = 1192), the association with sibling status was just short of conventional alpha (OR = 1.6, 95% CI: 1.0–2.7, p<0.056), similar to additional adjustment for other confounders: OR = 1.7, 95% CI: 1.0–2.9, p<0.053).

When conducting the analyses in the subsample of families without patients with OCD (exclusion of 91 patients, i.e. 9.2% of the patient group), the association between OCS and sibling status remained significant for broadly defined and clinically relevant OCS, even when adjusted for subclinical psychotic symptoms and demographical confounders (Table 3).

**Table 3:** Associations between OCS and sibling status psychosis liability.

Siblings vs. controls		
SAMPLE	Level of expression of OC phenotype	OR (95% CI:), p
All families in whole sample	Broad OCS	
	Adjusted for SIS-R pos. and neg. symptoms	1.7 (1.0–2.6); p<0.03
	Additionally adjusted for confounders *	1.8 (1.1–2.9), p<0.02
	Clinically relevant OCS	
	Adjusted for SIS-R pos. and neg. symptoms	2.7 (1.3–5.9), p<0.01
	Additionally adjusted for confounders *	2.8 (1.3–6.2), p<0.01
Within fam. without OCD pts.	Broad OCS	
	Unadjusted	1.8 (1.1–2.8), p<0.01
	Adjusted for SIS-R pos. and neg. symptoms	1.6 (1.0–2.6), p<0.04
	Additionally adjusted for confounders *	1.7 (1.0–2.8), p<0.03
	Clinically relevant OCS	
	Unadjusted	2.5 (1.2–5.5). p<0.01
	Adjusted for SIS-R pos. and neg. symptoms	2.7 (1.3–5.9), p<0.01
	Additionally adjusted for confounders *	2.4 (1.1–5.5), p<0.03

Controls = reference = 0; sibling of patient = 1

Associations are from multilevel logistic regression analysis, expressed in odds ratios; OR (95% CI:), p

### Cross-sib within trait associations

The cross-sib within-trait analysis revealed that broadly defined OCS (Y-BOCS >0) in patients were significantly associated with those of their related sibling (OR = 1.9; 95% CI: 1.1–3.1; p<0.014). When adjusted for age, sex, IQ, level of education, marital status and use of antipsychotics and antidepressants, effect sizes and specificity did not change substantially (OR = 1.9 (1.1–3.1); p<0.017). Similarly, clinically relevant OCS (Y-BOCS >9) in patients were significantly associated with those of their related siblings (OR = 2.6, 95% CI: 1.4–5.0, p = 0.004; adjusted OR = 2.3 (1.2–4.7), p<0.02).

### Cross-sib cross-trait associations

In the cross-sibling cross-trait analysis, the broadly defined OCS (Y-BOCS >0) in the patients were at trend level associated with the subclinical positive psychotic symptoms in their unaffected siblings, assessed with the SIS-R (Table 4). When adjusted for sex, IQ, level of education and marital status in sibs and patients, the specificity of the association reached conventional alpha. When additionally adjusted for age, it lost specificity (Table 4). Clinically relevant OCS (Y-BOCS >9) in patients were not significantly associated with the positive subclinical psychotic symptoms in their siblings, although directionally similar to the broadly defined OCS (Table 4).

**Table 4:** Multilevel linear regression estimates with sibling SIS-R scores for positive psychotic experiences as dependent variable and patient dichotomous YBOCS scores (> = 16 vs. <16) as independent variable.

Level of expression of OC phenotype	B (95% CI), p
Broad OCS	0.5 (-0.02–0.95), p<0.06
Adjusted demo (not age)*	0.5 (.02–1.0), p<.04
Adjusted demo (incl. age)**	0.5 (-0.04–0.95), p<.07
Adjusted demo + pharma***	0.4 (-0.1–0.9), p<0.2
Clinically relevant OCS	0.4 (-0.1–0.9), p<0.1
Adjusted demo (not age)*	0.4 (-0.1–0.9), p<0.1
Adjusted demo (incl. age)**	0.4 (-0.1–0.9), p<0.1
Adjusted demo + pharma***	0.3 (-0.28–0.8), p<0.4

Associations from multilevel linear regression equations, expressed as B (95% CI), p

* Adjusted for confounders sex, level of education, IQ, marital status,

**Additionally adjusted for age

**Additionally adjusted for use of antidepressants, use of antipsychotics.

Neither broadly defined nor clinically relevant OCS in patients showed a significant association with subclinical negative psychotic symptoms in unaffected siblings.

## Discussion

To our knowledge, this study is the first to demonstrate (i) an association between OCS and familial psychosis liability, independent of the phenotypical expression of subclinical psychotic symptoms, (ii) clustering of OCS within families of patients with psychotic disorder, and (iii) familial clustering of subclinical positive psychotic symptoms with OCS. Taken together, the findings are suggestive of a shared, possibly genetic, etiological mechanism for psychosis and OCS.

The first main finding in the current study is a significantly higher rate of OCS in patients with non-affective psychotic disorder and their unaffected siblings, compared to controls. These OCS were also significantly associated with psychosis liability, independent of the expression of (subclinical) psychotic symptoms. These associations were present for broadly defined OCS, and somewhat more pronounced in more narrowly defined “clinical” OCS. The prevalence rate of 19% of clinically relevant OCS in the patients diagnosed with non-affective psychotic disorder is in line with previously reported rates [9, 39, 40]. Only a few studies have assessed OCS in siblings of patients with psychosis. The rate of 4.3% of clinically relevant OCS we found in the siblings is in line with one earlier family study on OCD in relatives of probands with psychotic disorder, though data cannot be compared exactly. Poyurovsky et al. (2005) found a rate of 6.6% of clinical OCD in relatives of probands with “OCD schizophrenia”, 1.4% in relatives of probands with “non-OCD-schizophrenia”, and 0.7% in relatives of controls [18]. The difference between non-OCD-schizophrenia-relatives and controls was not significant. However, with a number of 210 relatives of 60 non-OCD-schizophrenia probands, the analyses may have been underpowered. The current study examined a considerably larger sample and found rates of clinically relevant OCS (YBOCS>9) of 10.3% in siblings of patients with “OCD-psychotic disorder”, compared to 4.0% in siblings of “non-OCD-psychotic disorder” patients and 1.6% in controls. The latter difference was significant, using a t-test (p<0.01). Moreover, clinically relevant OCS in unaffected sibs of patients with non-OCD psychotic disorder were significantly associated with psychosis liability indexed by sibling status. These findings are suggestive of a shared etiological pathway of the psychosis and OCD phenotype, independent of disease-related factors.

Essentially, Poyurovsky et al. (2005) showed that the familial clustering of the OCD phenotype that exists in the general population also exists in families with a member with diagnosis of schizophrenia. However, whether the OCD phenotype clusters with the psychosis phenotype, was not investigated in that study. Moreover, though the data may be compatible with an a priori defined “schizo-obsessive” diagnostic category, they do not support the validity of such a new diagnostic category.

It is of note that OCS in patients were not associated with subclinical negative symptoms in their unaffected sibling. This differential association of OCS with the positive, not negative symptom factor in psychosis, is in line with earlier findings [41]. However, in other work, no difference in positive or negative symptoms was found between a group with psychosis with OCD versus without OCD [40, 42, 43].

Second, we demonstrated that the OCD extended phenotype clusters within families of patients with psychosis, as it does in the general population. It has been shown earlier that an OCD phenotype, extending beyond the diagnostic threshold, can be measured in the general population, and that this OCD phenotype clusters within families, similar to the psychosis extended phenotype [20, 21, 44, 45]. The data in the current study show that the distributions of the extended phenotypes are preserved in this sample. This confirms the validity of the definition of the phenotype, even at subthreshold level and in the presence of co-occurring symptoms of other phenotypes. Furthermore, these findings show that the observed co-occurrence cannot just be explained by treatment bias or invalid diagnostic definitions.

Third, it was shown that OCS in patients were, at trend level, associated with subclinical positive psychotic symptoms in their unaffected siblings. When adjusted for demographical confounders other than age in patients as well as siblings, the association became significant suggesting familial clustering of subclinical positive psychotic symptoms with broadly defined OCS.

When additionally adjusted for age, the associations lost specificity. However, as the *a priori* determined demographical confounders were present in the subjects with the dependent as well as in those with the independent variable, a straightforward hypothesis about the expected potential impact of the variables selected as confounders cannot readily be made. In our analysis, there was no significant difference in age between sibs and patients. The role of age is particularly hard to predict, given the fact that OCD had a bimodal distribution of age of onset, and mean age of onset of OCD is expected to be lower than the mean age of onset of psychosis [46]. Therefore, the role of age as a confounder in this analysis should be interpreted cautiously [47, 48]. Apart from these included confounders, there may well be environmental confounding factors shared in families, that were not mapped in the current analysis, as demonstrated in a recent large study [19].

Despite a large sample, the cross-sib cross-trait analysis may have been underpowered. Strikingly, the effect size (ß = 0.5) of the association between OCS in patients and subclinical positive psychotic symptoms in unaffected relatives showed little variation in the models with broadly or more narrowly defined OCS, with or without confounders. The specificity however, was lower in the models with more stringently defined (“clinical”) OCS, probably due to lower power consequent to lower numbers.

Theoretically, cross-sib cross-trait analyses within the group of unaffected sibs would provide an elegant way to investigate an etiological association between the (subclinical level of expression of the) two extended phenotypes, ruling out disease-related factors more thoroughly. However, the number of sibs (158 pairs) proved too low to conduct these analyses.

The combination of (i) the association of OCS with familial psychosis liability in the whole sample, (ii) the preservation of familial clustering of the OCD phenotype within families with psychosis liability and (iii) the association, within families, between OCS in patients diagnosed with schizophrenia and subclinical positive psychotic symptoms in their unaffected sibs, may find its origin in a shared etiological, possibly genetic mechanism. The higher than chance cross-sectional and longitudinal co-occurrence of the psychosis and OC extended phenotype extensively described in earlier work [49, 50] fits well with this hypothesis. The associations found in the current study should be considered as compatible with, but certainly not a proof of a shared genetic etiology.

### Strengths and limitations

A strength of this study is the design with patients and their siblings in the same family, and an additional control group, in a large sample. The investigation of symptoms, including the lower end of the distribution of expression of the phenotype in unaffected siblings, allows ruling out the role of disease-related confounders. In addition, the hierarchical familial clustering of the data, and particularly the cross-sib cross-trait analyses, also enables to resolve the issue of disease- and treatment-related confounding and within-person confounding.

There are however some limitations. Some other mechanisms than those discussed above may contribute to the associations found.

First, the divergent (construct) validity of the concepts of positive psychotic symptoms and obsessions may be insufficient [51, 52]. Delusions and obsessions share some features in their conceptualisation: both concern persistent thoughts or ideas that become overvalued and go together with elevated preoccupation, distress, anxiety and, frequently, impact on behaviour [53, 54]. Although obsessions are typically “ego-syntonic” and accompanied with insight, whereas delusions are not, the differences in the population are gradual, as implied in the specifier “with poor insight” in the OCD criteria of the DSM IV[27], yielding possible overlap between the concepts. As a consequence, the association between OCS and positive subclinical psychotic symptoms may be spurious to the extent the concepts overlap. On the other hand, in an attempt to overcome the potential overlap between OC and psychosis symptoms as mentioned above, obsessions were defined as distressful thoughts not related to the subject’s psychotic symptoms, with full insight, and compulsions were defined as repetitive goal directed rituals clinically distinguishable from schizophrenic mannerisms or posturing. These definitions reduce the probability of misdiagnosing delusions for obsessions, however they may result in an underestimation of OCS [55, 56].

Second, in the investigation of the association between expressed OCS and psychosis liability (siblings versus controls), the role of a familial OCD liability that may exist independent of psychosis liability, is not completely excluded. However, by exclusion of the families with patients with a diagnosis of OCD, the role of such an OCD liability is reduced.

Third, use of antidepressants in patients apparently reduced the specificity of the association in the cross-sib cross-trait analyses. Most likely, the use of antidepressants is a proxy for the presence of OCS and affective symptoms in patients. The first-choice treatment of OCD consists of antidepressants. Thus, use of antidepressants most likely is an epiphenomenon.

Fourth, it was shown in earlier work that antipsychotics are associated with the induction of OCS [17, 57]. In the GROUP sample, (only) clozapine used for a period of over 6 months showed an association with OCS [58]. As a consequence, the decrease in specificity of the association between OCD in patients and subclinical psychotic symptoms in siblings, may be attributable to confounding.

The possibility of a partially shared etiological mechanism may have consequences for clinical practice, particularly as there is evidence that the course and outcome of co-occurring OCS and psychosis is unfavorable [59]. Therefore, co-occurrence of OCS with psychosis, particularly if persisting [49], may be reason for intensified monitoring. Further research is needed to further disentangle and understand the possible etiologic association of OCD and psychosis.
